# Hemophagocytic lymphohistiocytosis followed by an episode of peritoneal dialysis associated peritonitis: a case report

**DOI:** 10.1186/s12882-019-1217-1

**Published:** 2019-01-29

**Authors:** Bixia Gao, Xiaoyu Jia, Jicheng Lv, Jie Dong

**Affiliations:** Renal Division, Department of Medicine, Peking University First Hospital; Peking University Institute of Nephrology, Key Laboratory of Renal Disease, Ministry of Health of China; and Key Laboratory of Chronic Kidney Disease Prevention and Treatment (Peking University), Ministry of Education, 8 Xishiku Street; Xicheng District, Beijing, China

**Keywords:** Hemophagocytic lymphohistiocytosis, Peritoneal dialysis associated peritonitis

## Abstract

**Background:**

Hemophagocytic lymphohistiocytosis (HLH) is characterized by excessive activation of the immune system due to infection, autoimmune diseases, or malignancy. As an aggressive and life-threatening clinical syndrome, HLH secondary to peritoneal dialysis associated peritonitis (PDAP) has never been reported.

**Case presentation:**

A 34-year-old female peritoneal dialysis (PD) patient was hospitalized for fever, progressively multi-organ damage (including cytopenias, abnormalities of coagulation and liver enzyme) after an episode of organism-specific peritonitis. She was refractory to the broad-spectrum antimicrobial agent. Further tests found hemophagocytosis on the bone marrow examination, and extremely high level of sIL2-R and impaired activity of NK cell. The diagnosis of HLH was eventually established. After HLH-specific therapy, this patient recovered and discharged.

**Conclusions:**

The present case suggests that clinicians should to be aware of HLH in those patients apparently suspected with refractory or relapsing peritonitis, especially those accompanied with persist fever, hyperferritinemia, and cytopenias. HLH-specific therapy and supportive care should be applied without delay.

## Background

Peritoneal dialysis associated peritonitis (PDAP) is a common complication in peritoneal dialysis (PD) patients. About 10–20% of episodes would result in treatment failure including peritonitis-related death and transfer to hemodialysis. Also, severe or recurrent peritonitis is recognized to be associated with ultrafiltration failure and encapsulating peritoneal sclerosis (EPS). Hemophagocytic lymphohistiocytosis (HLH), as an aggressive and life-threatening clinical syndrome [1, 2], has never been reported in PD patients with or after an episode of peritonitis. This disorder is characterized by excessive activation of the immune system due to infection, autoimmune diseases, or malignancy [3], and leads to uncontrolled hypercytokinemia and multi-organ dysfunction [4, 5].

In this report, we described a female PD patient suffered from an episode of peritonitis. Her symptom was once clinically improved after anti-infective therapy, then developed fever and subsequently progressive multi-system damage. HLH was suspected and treatment was promptly initiated. After HLH-specific therapy, this patient recovered and was discharged.

## Case presentation

A 34-year-old Asian woman presented to the nephrology department of Peking University First Hospital in August 2015 with an over two-week history of intermittent fever. She had been on continuous ambulatory peritoneal dialysis for 9 months before admission. The patient had type 2 diabetes mellitus and initiated insulin injection five years before. Four months before admission the hemoglobin A1c level was 6.2%. Besides, she was diagnosed as anti-neutrophil cytoplasmic antibody (ANCA) associated glomerulonephritis three years before and treated with immunosupressive therapy of corticosteroid, cyclophosphamide and azathioprine. Except for predinisone with a dosage of 2.5 mg (mg) daily, other immunosuppressive agents had been discontinued one year before. She did not smoke, drink alcohol, or use illicit drugs. Her mother had diabetes mellitus too.

Fifteen days before admission, this patient had suffered from a fever of 37.5–38 degrees Celsius, and abdominal pain. Culture of cloudy peritoneal fluid with a high nucleated cell count of 1848/m^3^ (80% polymorphonuclear cells (PMNs)) grew Acinetobacter baumanni. She was diagnosed as PDAP and treated with intraperitoneal vancomycin (1 g every five day) and oral moxifloxacin, Clinically improvement was observed within 24 h. Peritoneal effluent became clear and nucleated cell count decreased to 10/m^3^ within five days. One week before admission, the patient presented to our emergency room with a high fever (39–40 degrees Celsius) again. She reported with nausea and anorexia, but without significant respiratory or abdominal symptoms. Initial laboratory tests showed significantly elevated C-reactive protein (CRP, 114 mg/L; reference range < 8 mg/L) and procalcitonin (PCT, 19.68 ng/mL; reference range < 0.05 ng/mL). A diagnosis of relapsing peritonitis was naturally suspected. Antibiotic therapy of intravenous meropenem and moxifloxacin were given immediately according to the antimicrobial susceptibility results of the last episode of PDAP. However, the patient did not respond to the antibiotic therapy. Clinical worsening was evident with a persistent fever (> 38 degrees Celsius) and symptoms of heart failure including dyspnea and chest distress.

Infectious causes of fever were thoroughly sought. Repeated exam of the peritoneal fluid nucleated cell count and PMNs showed no abnormal. No signs of bacteria, fungi or tuberculosis were found in the peritoneal fluid. Repeated cultures of peritoneal fluid and blood came back negative. A panel of respiratory viral antibodies were screened and no significant positive results were shown. Hypae of Candia albicans in the induced sputum was found. Chest computer tomography (CT) without contrast presented large areas of lung consolidation and ground-glass opacification. Thus, pulmonary fungal infection was suspected and oral voriconazole was added, although the bronchoscopy later found no significant inflammation and culture of bronchoalveolar lavage fluid (BALF) came back negative including fungi. Immunological tests showed negative ANCA, The levels of immunoglobulins and complement components had no obvious abnormalities. Abdominal and pelvic CT with contrast had no significant findings. The serological tumor markers were unremarkable.

On admission, the vital signs were as follows: temperature 38 degrees Celsius, respiratory rate 20 breaths/minute, blood pressure 130/80 mmHg, saturating 94% with supplementary oxygen through a nasal cannula at 4 L/min. Physical examination found no skin rash or enlarged lymph node. Breath sound was lower in both lungs. On abdominal exam, she was found abdominal distention with weak bowel sounds probably due to hypokalemia (serum potassium 2.0 mmol/L), but no hepatosplenomegaly. There was mild pitting edema bilaterally in the lower limbs. The patient switched to continuous vena-venous hemofiltration (CVVH) and the symptoms of heart failure improved. However, fever persisted without any response to antibiotic therapy. Meanwhile, we observed the pancytopenia, progressive decrease of fibrinogen, as well as elevated liver enzymes (Table 1).

**Table 1:** Laboratory data

	5 days before admission	On admission	3 days after admission*	7 days after admission	14 days after admission	Before discharge	Reference range
White-cell count (per mm^3^)	4700	2900	2700	2100	4200	5200	3500–9500
Hemoglobin (g/dL)	8.5	8.6	7.3	10.8*	8.2	10.1	13–17.5
Neutrophil (per mm^3^)	4260	2400	2330	1200	3200	3400	1800–6300
Platelets (per mm^3^)	123,000	75,000	60,000	34,000	91,000	155,000	125,000–350,000
Alanine aminotransferase (U/liter)	15	69	75	156	25	10	7–40
Fibrinogen (g/L)	3.34	2.4	2.05	1.05	1.72	2.22	2–4
C reactive protein (mg/L)	114	80	79.4	16.8	4.33	2.1	< 8
Procalcitonin (ng/ml)	19.68	5.243	/	0.975	0.338	0.136	< 0.05
Ferritin (ng/ml)	/	15,043	/	4729	1177.3	416	11–306

*Initiation of intravenous methylprednisolone

#After transfusion of red blood cells

**Fig. 1:**
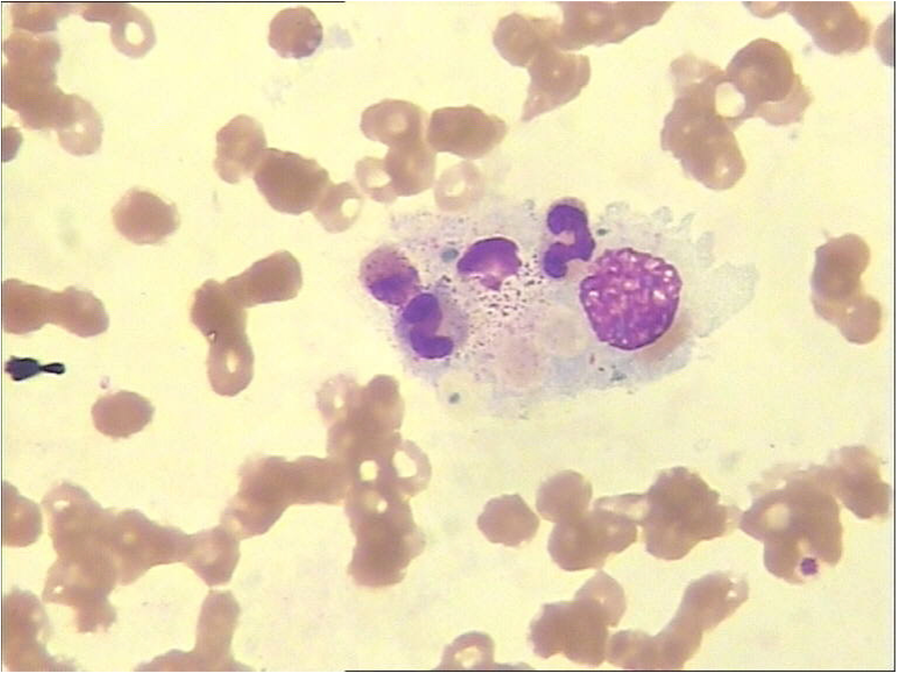
The bone marrow showed the presence of hemophagocytosis

Pulmonary infection was initially suspected but was later ruled out. Firstly, the patient had no specific symptoms of pneumonia, including coughing and sputum. The symptoms of dyspnea and chest distress were probably due to the acute heart failure, since these symptoms improved soon after CVVH therapy. Secondly, repeated chest CT (five days after the first chest CT) showed both lung consolidation and infiltration mostly absorbed. This dramatic change of chest image was more likely caused by heart failure, rather than pulmonary infection. Drug fever was also considered since the patient had administrated variety antimicrobial agents during the period. However, drug fever could not explain the deterioration of the clinical status and lab index. Further biochemical serum tests showed highly elevated ferritin (15,043.2 ng/ml). A bone marrow aspiration showed the presence of hemophagocytosis (Fig. 1). The HLH was suspected. Further molecular testing indicated low natural killer cell activity (11.16%, reference range 15.1–26.9%) and high levels of soluble interleukin-2 receptor (sIL2R, > 44,000 pg/mL, reference range < 6400 pg/mL), which added to fever, cytopenia, hypofibrinogenemia, increased ferritin and the presence of hemophagocytosis in bone marrow, met the criteria of HLH [6].

**Fig. 2:**
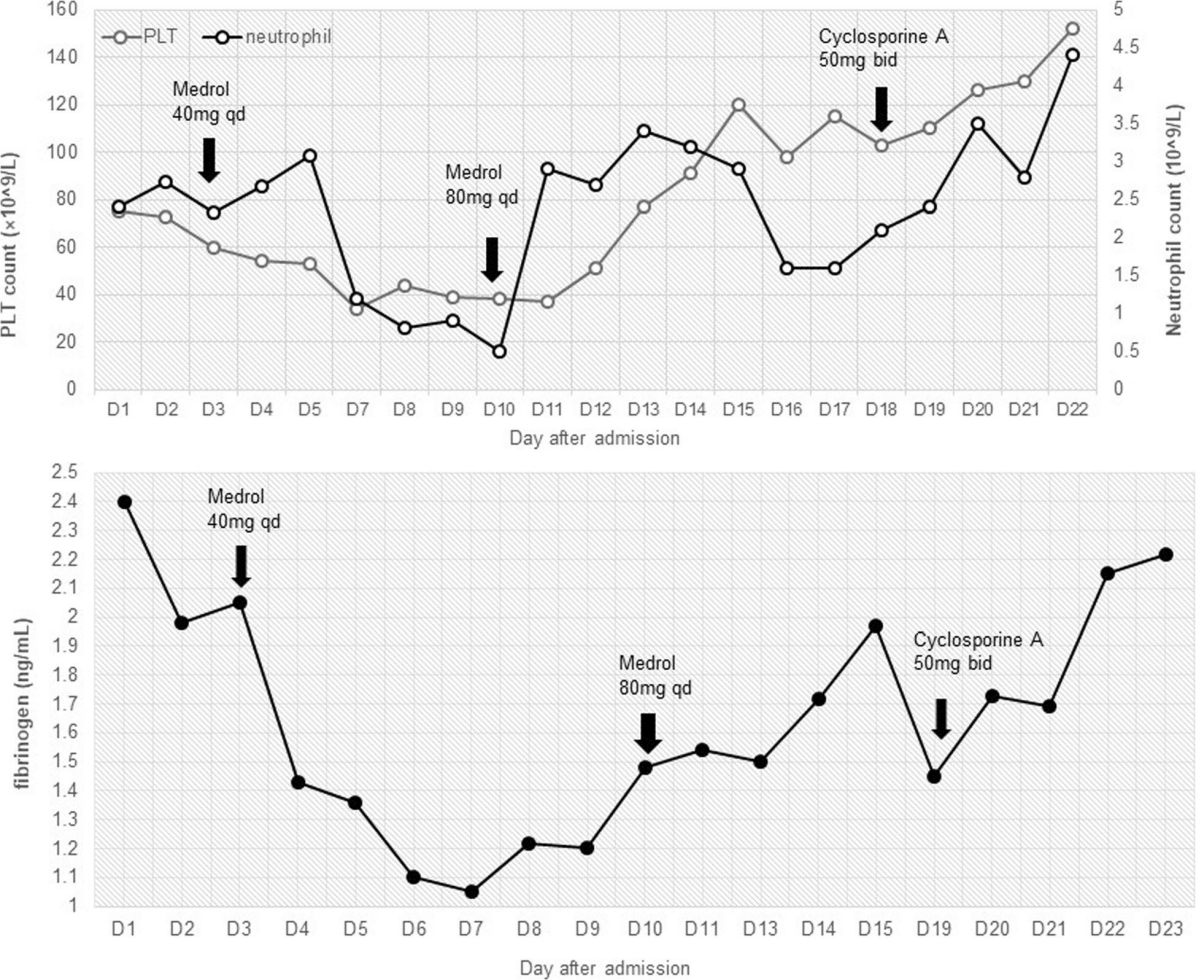
The trend graph of hemogram and fibrinogen after admission. The count of Platelets (PLT) (Gray curve) and neutrophil (Black curve) were showed in the upper panel; The level of fibrinogen was showed in the lower panel. Treatments include the initiation of intravenous methylprednisolone (Medrol), the dosage increase of Medrol, and the initiation of oral cyclosporine (arrows). PLT: platelets; Medrol: methylprednisolone

Intravenous methylprednisolone (Medrol) was administrated at a dosage of 40 mg daily. The general condition of the patient improved dramatically including the disappearance of nausea and abdominal distention. Fever subsided after the first dose of Medrol and the body temperature remained normal afterwards. The levels of CRP and PCT gradual dropped to normal range (Table 1). However, lab results still showed rapid decline of neutrophil count (minimum 0.5 × 10^9^/L), PLT count (minimum 34 × 10^9^/L), and fibrinogen (minimum 1.05 g/L) during the next week (Fig. 2), whereas the other coagulation parameters (prothrombin time, activated partial thromboplastin time) were all within the normal range. The dosage of Medrol increased to 40 mg twice a day, and cyclosporine was then administrated with target plasmatic levels between 100 and 200 μg/L, together with supportive treatment of intravenous immunoglobulin, fibrinogen, and fresh frozen plasma transfusion. We observed a gradual improvement of cytopenia and coagulopathy (Fig. 2). The patient was discharged and continued the maintain-phase treatment of HLH on a regimen of oral prednisolone and cyclosporine. The full course of glucocorticoid and cyclosporine was 12 weeks referring to the HLH-2004 protocol [7]. After discharge, the patient went on intermittent hemodialysis in our hemodialysis center. The laboratory tests were taken every 3–6 months. The latest lab results (June 2018) showed normal complete cell count (white-cell count 5830/mm^3^, hemoglobin 11.5 g/dL, platelets 138,000/mm^3^). The levels of ferritin and CRP were also in the normal range (123.5 ng/ml and 3.04 mg/L, respectively). Until now, no recurrence of HLH was observed.

## Results and conclusions

To the best of our knowledge, we report the first case of HLH following an episode of PDAP. Our case suffered from an episode of organism-specific peritonitis shortly before the exacerbation of illness. No signs of relapse of peritonitis and acute abdomen complications were observed after the recurrence of fever. She was refractory to the broad-spectrum antimicrobial agents with presentations of hyperferritinemia, and progressively multi-organ damage including cytopenias, abnormalities of coagulation and liver enzyme. Hemophagocytosis was detected on the bone marrow examination. Those signs made the clinical suspicion of HLH. Further molecular testing found the extremely high level of sIL2-R and impaired activity of NK cell. The diagnosis of HLH was eventually established according to the HLH-2004 protocol [7].

The HLH is broadly divided into primary HLH and acquired HLH [8]. Primary HLH mainly affects infants, which is caused by genetic mutations impairing the cytotoxic function of natural killer and cytotoxic T cells [9, 10]. Acquired HLH generally affects adolescents and adults and has remarkable increased incidence over the past decade [11]. Infection, especially Epstein-Barr viral infection, is a common trigger of acquired HLH [12]. Although less common, HLH which occurs in patients with bacterial infection has also been reported [11, 13]. Our case developed HLH during the therapy of an episode of bacterial peritonitis. Further extensive assess excluded other infectious pathogens, the relapse of AASV, underlying malignancy especially lymphoma, as well as drug fever, which were potential triggers of developing HLH in our patient. Finally, we speculate that this episode of PDAP acted as a trigger of developing HLH. In addition to the external triggers, genetic defect has also been reported in few adult HLH patients [14, 15]. Whether our case had the genetic predisposition of developing HLH is unclear. The patient declined the genetic testing due to the expensive cost.

In HLH patients, the uncontrolled activated CTLs, NK cells, and macrophages result in the excessive secretion of multiple inflammatory cytokines, in terms of “cytokine storm”, and eventually lead to the tissue damage and multi-organ failure [4, 16]. Clinically, sIL2-R and NK function are important objective markers of increased T cell activity and impaired cytotoxic function, respectively. Whereas the notably elevated ferritin level reflects the excessive activation of macrophages and hypercytokinemia. Early recognition, immediately intervention and search for the underlying triggers are essential for the outcome of those HLH patients. In the phase of initial therapy, the purpose it to control the overactive immune system and to halt the underlying trigger. Glucocorticoid is always included as the first-line regimens, irrespective of the cause. Other immunosuppressive agents are selectively involved, including etoposide, cyclosporine, methotrexate, cyclophosphamide [11, 12]. Supportive care is as essential for the opportunistic infection, coagulopathy, as well as multiple organ damage [5, 11]. In the infection-associated HLH, the intravenous immunoglobulin (IVIG) is clinically preferred [17].

In our case, the intravenous Medrol was initiated immediately after admission and the temperature dropped to normal dramatically on the first day of Medrol. However, progressive cytopenias, liver function abnormality, and coagulopathy continued. Since further assessment confirmed the diagnosis of HLH, we speculated that the uncontrolled immune activation of HLH had not been completely suppressed by the Medrol alone. Thus, the dose of Medrol was doubled and cyclosporine was then added. In addition, supportive cares including transfusions of blood components, IVIG, and CVVH were simultaneously implemented. After above treatment strategies, the damage of multi organs gradually improved. Subsequently, the plasma levels of ferritin and sIL2-R significantly declined 4 weeks after the initiation of HLH-specific therapy (sIL2-R 8493 pg/mL (reference range < 6400 pg/mL) and ferritin 416 ng/ml; respectively). Without therapy, the mortality of patients with HLH is high, especially for the primary HLH. For the adult HLH, the prognosis were heterogeneous. One retrospective analysis described 162 adult patients and reported that the overall mortality was 42% [2]. Mortality rate was generally lowest in autoimmune diseases followed by infection-associated HLH [18]. Malignancy-associated HLH (especially T cell lymphomas) was proven one prominent adverse prognosis marker. Other risk factors included older age, as well as some markers of disease severity (e.g, decreasing platelet count, highly elevated ferritin) [18]. For our patients, earlier diagnosis and timely disease-specific immunotherapies might explain the benign prognosis.

In our patient, the HLH developed after the improvement of an episode of peritonitis. It was speculated that the cytokine cascade in the process of HLH may not be inhibited despite the infectious trigger has been controlled. This feature was similar to the development of peritoneal dialysis related EPS, which was a rare but severe complication of peritoneal dialysis characterized by progressively peritoneal thickening, intraperitoneal fibrosis, and encasement of bowel loops [19]. The peritoneal inflammation and fibrosis of EPS could not be stopped by the cure of peritonitis [20]. The underlying pathogenesis of HLH in PDAP patients need to be searched in the future.

To our knowledge, HLH secondary to an episode of PDAP has not been reported yet. Clinicians should to be aware of HLH in those patients apparently suspected with refractory or relapsing PDAP, especially those accompanied with persist fever, hyperferritinemia, and cytopenias. HLH-specific therapy and supportive care should be applied without delay.

## Abbreviations

ANCA: anti-neutrophil cytoplasmic antibody
BALF: bronchoalveolar lavage fluid
CRP: C-reactive protein
CT: computer tomography
CVVH: continuous vena-venous hemofiltration
EPS: encapsulating peritoneal sclerosis
HLH: hemophagocytic lymphohistiocytosis
PCT: procalcitonin
PD: peritoneal dialysis
PDAP: peritoneal dialysis associated peritonitis
PMNs: polymorphonuclear cells
sIL2-R: soluble interleukin-2 receptor

## References

[1] Janka GE (1983). Familial hemophagocytic lymphohistiocytosis. Eur J Pediatr.

[2] Riviere S, Galicier L, Coppo P, Marzac C, Aumont C, Lambotte O, Fardet L (2014). Reactive hemophagocytic syndrome in adults: a retrospective analysis of 162 patients. Am J Med.

[3] Schram AM, Berliner N (2015). How I treat hemophagocytic lymphohistiocytosis in the adult patient. Blood.

[4] Filipovich A, McClain K, Grom A (2010). Histiocytic disorders: recent insights into pathophysiology and practical guidelines. Biol Blood Marrow Transplant.

[5] Hayden A, Park S, Giustini D, Lee AY, Chen LY (2016). Hemophagocytic syndromes (HPSs) including hemophagocytic lymphohistiocytosis (HLH) in adults: a systematic scoping review. Blood Rev.

[6] Jordan MB, Allen CE, Weitzman S, Filipovich AH, McClain KL (2011). How I treat hemophagocytic lymphohistiocytosis. Blood.

[7] Henter JI, Horne A, Arico M, Egeler RM, Filipovich AH, Imashuku S, Ladisch S, McClain K, Webb D, Winiarski J (2007). HLH-2004: diagnostic and therapeutic guidelines for hemophagocytic lymphohistiocytosis. Pediatr Blood Cancer.

[8] Filipovich AH (2011). The expanding spectrum of hemophagocytic lymphohistiocytosis. Curr Opin Allergy Clin Immunol.

[9] Chandrakasan S, Filipovich AH (2013). Hemophagocytic lymphohistiocytosis: advances in pathophysiology, diagnosis, and treatment. J Pediatr.

[10] Stepp SE, Dufourcq-Lagelouse R, Le Deist F, Bhawan S, Certain S, Mathew PA, Henter JI, Bennett M, Fischer A, de Saint Basile G (1999). Perforin gene defects in familial hemophagocytic lymphohistiocytosis. Science.

[11] Ramos-Casals M, Brito-Zeron P, Lopez-Guillermo A, Khamashta MA, Bosch X (2014). Adult haemophagocytic syndrome. Lancet.

[12] Rouphael NG, Talati NJ, Vaughan C, Cunningham K, Moreira R, Gould C (2007). Infections associated with haemophagocytic syndrome. Lancet Infect Dis.

[13] Risdall RJ, Brunning RD, Hernandez JI, Gordon DH (1984). Bacteria-associated hemophagocytic syndrome. Cancer.

[14] Ueda I, Kurokawa Y, Koike K, Ito S, Sakata A, Matsumora T, Fukushima T, Morimoto A, Ishii E, Imashuku S (2007). Late-onset cases of familial hemophagocytic lymphohistiocytosis with missense perforin gene mutations. Am J Hematol.

[15] Zhang K, Jordan MB, Marsh RA, Johnson JA, Kissell D, Meller J, Villanueva J, Risma KA, Wei Q, Klein PS (2011). Hypomorphic mutations in PRF1, MUNC13-4, and STXBP2 are associated with adult-onset familial HLH. Blood.

[16] Fujiwara F, Hibi S, Imashuku S (1993). Hypercytokinemia in hemophagocytic syndrome. Am J Pediatr Hematol Oncol.

[17] Morimoto A, Nakazawa Y, Ishii E (2016). Hemophagocytic lymphohistiocytosis: pathogenesis, diagnosis, and management. Pediatr Int.

[18] Arca M, Fardet L, Galicier L, Riviere S, Marzac C, Aumont C, Lambotte O, Coppo P (2015). Prognostic factors of early death in a cohort of 162 adult haemophagocytic syndrome: impact of triggering disease and early treatment with etoposide. Br J Haematol.

[19] Brown EA, Van Biesen W, Finkelstein FO, Hurst H, Johnson DW, Kawanishi H, Pecoits-Filho R, Woodrow G (2009). Length of time on peritoneal dialysis and encapsulating peritoneal sclerosis: position paper for ISPD. Perit Dial Int.

[20] Kawaguchi Y, Tranaeus A (2005). A historical review of encapsulating peritoneal sclerosis. Perit Dial Int.

